# Characterization of a highly stable mixed‐mode reversed‐phase/weak anion‐exchange stationary phase based on hybrid organic/inorganic particles

**DOI:** 10.1002/jssc.202001136

**Published:** 2021-01-18

**Authors:** Thomas H. Walter, Bonnie A. Alden, Jessica A. Field, Nicole L. Lawrence, Donna L. Osterman, Amit V. Patel, Maureen A. DeLoffi

**Affiliations:** ^1^ Waters Corporation Milford Massachusetts USA

**Keywords:** Atlantis BEH C18 AX, hybrid particles, mixed‐mode chromatography, mixed‐mode stationary phases, reversed‐phase/weak anion‐exchange

## Abstract

We have characterized Atlantis ethylene‐bridged hybrid C_18_ anion‐exchange, a mixed‐mode reversed‐phase/weak anion‐exchange stationary phase designed to give greater retention for anions (e.g., ionized acids) compared to conventional reversed‐phase materials. The retention and selectivity of this stationary phase were compared to that of three benchmark materials, using a mixture of six polar compounds that includes an acid, two bases, and three neutrals. The compatibility of the ethylene‐bridged hybrid C_18_ anion‐exchange material with 100% aqueous mobile phases was also evaluated. We investigated the batch‐to‐batch reproducibility of the ethylene‐bridged hybrid C_18_ anion‐exchange stationary phase for 27 batches across three different particle sizes (1.7, 2.5, and 5 μm) and found it to be comparable to that of one of the most reproducible C_18_ stationary phases. We also characterized the acid and base stability of the ethylene‐bridged hybrid C_18_ anion‐exchange stationary phase and the results show it to be usable over a wide pH range, from 2 to 10. The extended upper pH limit relative to silica‐based reversed‐phase/weak anion‐exchange materials is enabled by the use of ethylene‐bridged hybrid organic/inorganic particles. The improved base stability allows Atlantis ethylene‐bridged hybrid C_18_ anion‐exchange to be used with a wider range of mobile phase pH values, opening up a greater range of selectivity options.

**Article Related Abbreviations**
AMP: adenosine 5′‐monophosphate
AX: anion‐exchange
BEH: ethylene‐bridged hybrid
CSH: charged surface hybrid
HSS: high strength silica
TMP: thymidine 5′‐monophosphate

## 1 INTRODUCTION

Reversed‐phase chromatography has long been the most frequently used mode of HPLC for separations of small molecules [1, 2]. However, retaining and separating ionized polar acidic analytes in the RP mode is a challenge. The most widely used RP packing materials, those based on silica, typically exhibit cation exchange in addition to RP retention. This is due to the presence of residual silanol groups, which are acidic [3]. Ionized silanols create a negative surface charge, which repels negatively charged analytes such as ionized acids. Strategies to retain ionized acids include using ion‐pairing reagents in the mobile phase [4] or derivatization to form more hydrophobic neutral species [5]. These approaches have several disadvantages, including long equilibration times and high background levels when using ion‐pairing reagents and the greater time needed for sample preparation and the potential for increased variability with derivatization. Other chromatographic modes, including anion‐exchange [6] and hydrophilic interaction [7, 8], have been used for separating acidic compounds. However, ion‐exchange chromatography is not able to separate mixtures of acids, bases, and neutrals. Hydrophilic interaction chromatography, while providing excellent retention for ionized polar acids, suffers from challenges including finding a compatible sample solvent and long column equilibration times [9].

An alternative solution for retaining and separating ionized acids is to use a mixed‐mode stationary phase possessing both RP and anion‐exchange (AX) characteristics. Lämmerhofer et al. described the chromatographic characterization of mixed‐mode ion‐exchangers, including a number of commercially available products [10]. Mansour and Danielson reviewed mixed‐mode HPLC and its use for separations of small molecules [11]. Zhang and Liu summarized pharmaceutical applications of mixed‐mode HPLC and also discussed the structures of a number of commercially available mixed‐mode stationary phases [12]. Mixed‐mode materials reported by academic researchers have also recently been reviewed [13, 14].

Most of the commercially available mixed‐mode RP/AX columns are based on silane‐bonded silica stationary phases. Such bonded phases, particularly those that are synthesized using monofunctional dimethylorganosilanes, suffer from relatively poor stability when used with acidic mobile phases [15]. These bonded phases may also exhibit instability with basic mobile phases, due to dissolution of the silica particles [16]. Elution of bonded phase hydrolysis products containing amine groups from these stationary phases may interfere with ESI MS detection [17, 18]. Organic polymer‐based mixed‐mode columns have been reported to be stable over a wide pH range (e.g., 1–14), but suffer from low efficiencies [19]. Recent work to improve the hydrolytic stability of silica‐based mixed‐mode RP/AX materials used a polymer‐coating approach and functionalization via thiol‐ene click reactions [18]. The resulting stationary phase was shown to have good stability when used with a pH 5 mobile phase at 60°C.

To further improve hydrolytic stability without sacrificing column efficiency, a mixed‐mode RP/AX stationary phase based on ethylene‐bridged hybrid (BEH) organic/inorganic particles was recently developed, named Atlantis BEH C_18_ AX. Here, we describe the characterization of this material for retention and selectivity, column efficiency, compatibility with a 100% aqueous mobile phase, batch‐to‐batch reproducibility, and acid and base stability. The results are compared to those obtained for three RP stationary phases: BEH C_18_, high strength silica (HSS) T3, and charged surface hybrid (CSH) C_18_. These materials were chosen as benchmarks because they are widely used and share several characteristics with BEH C_18_ AX columns: the BEH particle chemistry (for BEH C_18_ and CSH C_18_) [20], the intermediate coverage C_18_ bonded phase (for HSS T3) [21], and the presence of both C_18_ and AX groups (for CSH C_18_) [22, 23].

## 2 MATERIALS AND METHODS

### 2.1 Chemicals

LC‐MS grade acetonitrile, MS grade formic acid, adenosine 5′‐monophosphate (AMP), and guanosine 5′‐monophosphate were obtained from Fisher Scientific (Hampton, NH). Methanol was obtained from Honeywell (Muskegon, MI). Ammonium formate and all other analytes were sourced from Millipore‐Sigma (Burlington, MA). Deionized water was produced using a Millipore Milli‐Q system.

### 2.2 Instrumentation and columns

All chromatographic evaluations were performed using ACQUITY UPLC Classic, H‐Class, or I‐Class instruments equipped with ACQUITY photodiode array detectors (Waters, Milford, MA). ACQUITY UPLC BEH C_18_, CSH C_18_, HSS T3, and Atlantis PREMIER BEH C_18_ AX columns (1.7 or 1.8, 2.5 and 5 μm, 2.1 × 50 mm) were obtained from Waters (Milford, MA).

### 2.3 Sample and mobile phase preparation

The sample used for the column efficiency test contained 10 μg/mL thiourea, 100 μg/mL naphthalene, and 200 μg/mL acenaphthene dissolved in acetonitrile/water (75:25, v/v.). The sample for the polar mixture separation contained 5 μg/mL thiourea, 12.5 μg/mL 5‐fluorouracil, 25 μg/mL nicotinamide, 37.5 μg/mL procainamide hydrochloride, 12.5 μg/mL AMP, and 125 μg/mL resorcinol in 10 mM ammonium formate pH 3.00 (aqueous). Void time (*t*_0_) measurements were made using thiourea with an acetonitrile mobile phase. The aqueous buffer used in the mobile phase was prepared by dissolving ammonium formate in water at a concentration of 10 mM, then adjusting the pH to 3.00 ± 0.01 using formic acid. For the study of the effect of mobile phase pH on retention, the 100% aqueous mobile phases were prepared from 20 mM ammonium formate with variable amounts of formic acid or ammonium hydroxide added to adjust the pH. The sample for the flow stop‐and‐restart experiment contained 10 μg/mL thiourea and 12 μg/mL thymine in 10 mM ammonium formate pH 3.00 (aqueous). Two samples were used for the accelerated acid stability study, one containing 25 μg/mL thiourea and 50 μg/mL methyl paraben in water and the other containing 25 μg/mL thiourea and 50 μg/mL thymidine 5′‐monophosphate (TMP) disodium salt hydrate in water. Two samples were also used for the accelerated high pH stability test: one containing 6 μg/mL uracil and 50 μg/mL propyl paraben, and the other containing 6 μg/mL uracil and 50 μg/mL TMP in methanol/water (30:70, v/v). The sample for the pH 10 stability study contained 10 μg/mL thiourea, 100 μg/mL guanosine 5′‐monophosphate disodium salt, 100 μg/mL resorcinol, and 100 μg/mL nicotinamide in water.

### 2.4 Method details

The column efficiency measurements were made for a 2.1 × 50 mm column packed with 1.7 μm BEH C_18_ AX. The mobile phase was acetonitrile/10 mM ammonium formate (75:25, v/v), pH 3.00, the temperature was 30°C, and UV absorbance detection (254 nm) was used. The sample contained thiourea as the *t*_0_ marker and acenaphthene as the retained analyte. Four sigma efficiencies were measured for acenaphthene. Reduced plate heights were calculated by dividing the plate heights by the nominal particle size [24]. The interstitial linear velocities were obtained from the ratio of column length to *t*_0_. The reduced linear velocities were calculated by dividing these values by the nominal particle size and the diffusion coefficient of acenaphthene in the mobile phase, 1.46 × 10^−5^ cm^2^/s, estimated using the Scheibel modification of the Wilke−Chang equation [25].

For the separations of the polar mixture, four consecutive isocratic separations were carried out using 2.1 × 50 mm columns with a 10 mM ammonium formate pH 3.00 (aqueous) mobile phase at 0.2 mL/min, an injection volume of 1.5 μL, a temperature of 30°C, and UV absorbance detection (254 nm). The retention times from the last three injections and the *t*_0_ measured using thymine with a 100% acetonitrile mobile phase were used to calculate the retention factors.

For the flow stop‐and‐restart experiment, a 1.5 μL injection of a sample containing thiourea and thymine was separated using 2.1 × 50 mm columns with a 10 mM ammonium formate pH 3.00 (aqueous) mobile phase at 0.2 mL/min and 30°C with UV absorbance detection (254 nm). The flow was stopped for 10 min, then restarted at 0.2 mL/min. After 0.6 min, two 1.5 μL injections of the thiourea/thymine sample were made. The retention factor of thymine was calculated using the retention time of thymine and the *t*_0_ measurement previously obtained using thiourea with a 100% acetonitrile mobile phase.

The accelerated acid stability test was carried out on 2.1 × 50 mm columns using a mobile phase containing 0.5% TFA (aqueous) at a flow rate of 1.4 mL/min, a temperature of 60°C and UV absorbance detection (254 nm). At 40 min intervals, 2 μL of a sample containing thiourea and methyl paraben was injected, followed by 2 μL of a sample containing thiourea and TMP, continuing for 24 h. Columns packed with two different batches of 1.7 μm BEH C_18_ AX were tested, along with one 1.8 μm HSS T3 column.

For the accelerated base stability test, two injections of a uracil sample were made on 2.1 × 50 mm columns to determine *t*_0_, then 2 μL of a sample containing uracil and propyl paraben, followed by 2 μL of a sample containing uracil and TMP. The mobile phase contained methanol/water (30:70 v/v) with 0.1% formic acid, the flow rate was 0.21 mL/min and UV absorbance detection (254 nm) was used. The mobile phase was then changed to 0.02 M NaOH (aqueous) and the flow rate was increased to 0.42 mL/min. After 60 min, the column was flushed with methanol/water (10:90, v/v), then methanol, both for 20 min at 0.21 mL/min. This entire sequence was repeated until a 50% decrease in efficiency or retention of propyl paraben was observed. The temperature was held at 50°C during this test. Columns packed with two different batches of 1.7 μm BEH C18 AX were tested, along with one 1.7 μm CSH C18 column.

For the pH 10 stability study, BEH C_18_ AX 1.7 μm 2.1 × 50 mm columns were used with a mobile phase of 10 mM ammonium bicarbonate pH 10.08 (aqueous)/methanol (95:5, v/v). The flow rate was 0.2 mL/min, the injection volume was 2 μL, the temperature was 30°C, and UV absorbance detection (254 nm) was used. The sample was injected 340 times at an interval of 60 min. Two columns were tested.

## 3 RESULTS AND DISCUSSION

### 3.1 Chemical and physical properties of the stationary phases

In Table 1 we detail the chemical and physical properties of the BEH C_18_ AX stationary phase as well as those of CSH C_18_, BEH C_18_, and HSS T3. The BEH particles used for the BEH C_18_ AX stationary phase have an average pore diameter of 95 Å, smaller than the 130 Å particles used for the BEH C_18_ and CSH C_18_ stationary phases. The reason for using the smaller pore size particles is for increased retention, stemming from the 46% higher surface area. BEH C_18_ AX columns give retention factors for neutral compounds that are very similar to those obtained using HSS T3 columns, which contain an intermediate‐coverage C_18_ bonded phase based on 100 Å silica particles. The BEH C_18_ AX stationary phase contains both C_18_ and tertiary alkylamine groups, the latter creating a positive surface charge below approximately pH 9 [26]. CSH C_18_ is similar to BEH C_18_ AX in having both C_18_ and anion‐exchange groups. However, the anion‐exchange groups in CSH C_18_ contain pyridine functionalities, which are positively charged only below approximately pH 5–6 [26, 27].

**TABLE 1:** Comparison of the chemical and physical properties of the stationary phases evaluated

	Particle properties^a)^				Bonded phase properties		
Stationary phase	Material	Average pore diameter (Å)	Pore volume (cm^3^/g)	Surface area (m^2^/g)	C_18_ surface concentration (μmol/m^2^)	Endcapping	Ion‐exchange group
BEH C_18_ AX	BEH	95	0.7	270	1.6	Yes	Alkylamine
CSH C_18_	BEH	130	0.7	185	2.3	Yes	Pyridyl
BEH C_18_	BEH	130	0.7	185	3.1	Yes	None
HSS T_3_	Silica	100	0.7	230	1.6	Yes	None

^a)^ The average pore diameter, pore volume, and surface area were determined for the unbonded particles using multipoint N_2_ sorption.

### 3.2 Column efficiency

**FIGURE 1:**
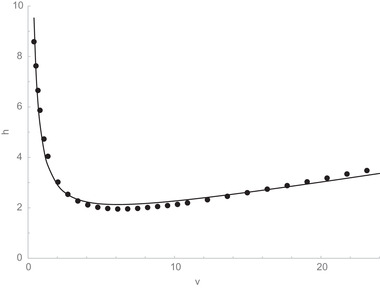
Reduced plate height (*h*) versus reduced linear velocity (*ν*) for a 2.1 × 50 mm column packed with 1.7 μm BEH C_18_ AX. The circles are the experimental values and the line is the best fit to the reduced van Deemter equation (*h* = 0.998 + 3.466/*v* + 0.093*v*). The retained analyte was acenaphthene, the mobile phase was acetonitrile/10 mM ammonium formate (pH 3.00) (75:25, v/v), and the temperature was 30°C

Columns packed with the BEH C_18_ AX stationary phase exhibit high efficiencies for neutral analytes, comparable to those of BEH C_18_, CSH C_18_, and HSS T3 columns. Shown in Figure 1 is a plot of reduced plate height (*h*) versus reduced linear velocity (*ν*) for a 2.1 × 50 mm column packed with 1.7 μm BEH C_18_ AX. The results show that a minimum reduced plate height of 2.06 was observed, modestly better than the values of 2.8 and 2.3 previously reported for 1.7 μm BEH C_18_ and BEH Shield RP_18_ columns [28].

### 3.3 Compatibility with 100% aqueous mobile phases

Highly aqueous mobile phases are often needed to obtain sufficient retention of polar analytes in RP separations. However, not all RP columns are compatible with such mobile phases, due to retention losses that may occur when the flow is stopped and restarted [29, 30]. Particularly prone to this phenomenon are high‐coverage C_18_ bonded phases based on particles with average pore diameters less than about 100 Å, for which retention losses approaching 100% have been reported. In an experiment in which the flow of a 100% aqueous mobile phase (at 30°C) was stopped and restarted for a 2.1 × 50 mm column packed with 1.7 μm BEH C_18_ AX, we observed only a 3.7% decrease in retention. This compares favorably to the 10.0% retention loss measured for a 1.8 μm HSS T3 column and the 8.2% loss found for a 1.7 μm BEH C_18_ column, and is similar to the 3.3% loss observed for a 1.7 μm CSH C_18_ column. The intermediate C_18_ surface concentration (1.6 μmol/m^2^) together with the hydrophilic anion exchange groups makes BEH C_18_ AX compatible with highly aqueous mobile phases.

### 3.4 Retention and selectivity

**FIGURE 2:**
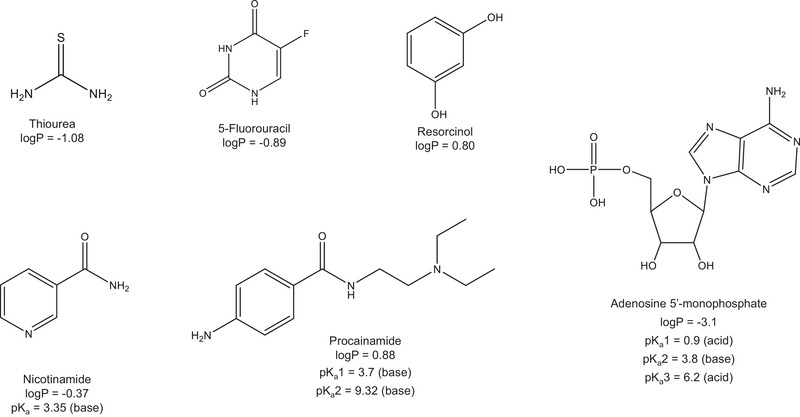
Structures and key properties of the test analytes

**FIGURE 3:**
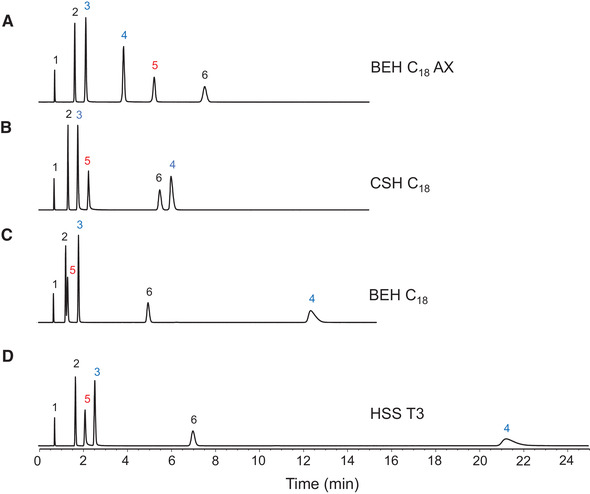
Isocratic separations of a mixture of polar analytes using four different columns. The mobile phase was 10 mM ammonium formate (pH 3.00) in water. Blue signifies compounds that are positively charged in the pH 3.00 mobile phase, red signifies the negatively charged compound, and black signifies the neutral compounds: 1: thiourea, 2: 5‐fluorouracil, 3: nicotinamide, 4: procainamide, 5: adenosine 5′‐monophosphate, 6: resorcinol. All columns were 2.1 × 50 mm. (A) BEH C_18_ AX, 1.7 μm, (B) CSH C_18_, 1.7 μm, (C) BEH C_18_, 1.7 μm, (D) HSS T3, 1.8 μm

To characterize the retention and selectivity of the BEH C_18_ AX stationary phase, we used a mixture of six polar compounds. The structures and key properties of these analytes are shown in Figure 2. With the exception of p*K*_a_1 for procainamide, the log*P* (log of the octanol/water partition coefficient) and p*K*_a_ values are experimental properties (pubchem.ncbi.nlm.nih.gov) [31]. The value for p*K*_a_1 for procainamide is an estimate based on the Hammett equation [32]. Three of the analytes (thiourea, 5‐fluorouracil, and resorcinol) are neutral in the pH 3 mobile phase, with log*P* values ranging from −1.08 to 0.80. AMP is negatively charged in the pH 3 mobile phase, and nicotinamide and procainamide are positively charged. The chromatograms obtained for a BEH C_18_ AX column and three benchmark columns are shown in Figure 3. For negatively charged AMP (peak 5), the BEH C_18_ AX column gave the highest retention. The neutral compounds (peaks 2 and 6) have similar retention on the BEH C_18_ AX and HSS T3 columns, while the BEH C_18_ and CSH C_18_ columns have lower retention of these analytes, primarily due to the lower surface area of the 130 Å particles used for the latter stationary phases. For the positively charged analytes nicotinamide and procainamide (peaks 3 and 4), the HSS T3 column gave the most retention, while the BEH C_18_ AX column gave the least.

The retention trends for the charged analytes may be explained by differences in the surface charge of the different stationary phases. The BEH C_18_ AX and CSH C_18_ stationary phases have a positive surface charge at pH 3 due to the presence of the anion‐exchange groups, with BEH C_18_ AX having the greater positive charge [26]. The positive surface charge results in increased retention of anions, such as AMP, and decreased retention of cations, such as nicotinamide and procainamide. The BEH C_18_ stationary phase exhibits a neutral surface charge under the test conditions, while the HSS T3 stationary phase appears to be negatively charged, due to the presence of ionized silanol groups. This causes decreased retention of AMP and increased retention of nicotinamide and procainamide. This is particularly evident for procainamide, which has a charge of +2 at pH 3, and thus exhibits pronounced ion‐exchange behavior. The positive surface charge of the BEH C_18_ AX and CSH C_18_ stationary phases also results in improved symmetry for the procainamide peak (peak 4) compared to the tailing peaks observed for the BEH C_18_ and HSS T3 columns [22, 27].

### 3.5 Batch‐to‐batch reproducibility

**FIGURE 4:**
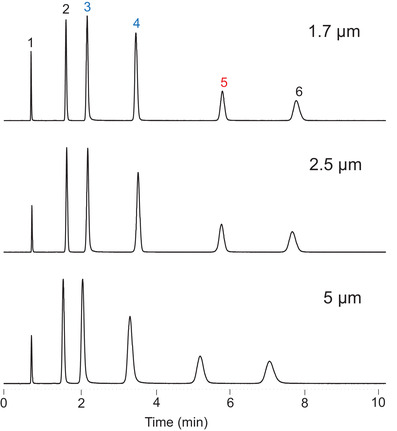
Separations of the mixture shown in Figure 2 using columns packed with three different batches of BEH C_18_ AX differing in particle size: top 1.7 μm, middle 2.5 μm, and bottom 5 μm. All columns were 2.1 × 50 mm. The chromatographic conditions were the same as in Figure 3

The surface modification procedure for BEH C_18_ AX involves separate steps to incorporate the anion‐exchange groups, the C_18_ groups and the endcap, similar to the approach used for CSH C_18_ [22, 23]. This gives good control of the surface chemistry, resulting in high batch‐to‐batch reproducibility. To assess the chromatographic reproducibility of BEH C_18_ AX, 27 different batches of 1.7, 2.5, and 5 μm materials were tested using the separation of the polar mixture shown in Figure 2. Representative chromatograms are shown in Figure 4. The results for the relative retentions (referenced to resorcinol) showed relative standard deviations (RSDs) ranging from 1 to 3%, comparable to one of the most reproducible conventional C_18_ bonded phases, Symmetry C_18_ [33]. The relative retentions for the different particle sizes were found to be statistically identical, facilitating scalability across columns packed with different particle sizes. Slight offsets in retention times which are consistent across all five retained compounds are due to the use of packing pressures that are optimized for the different particle sizes, resulting in nonidentical interstitial porosities of the columns.

### 3.6 Hydrolytic stability

**FIGURE 5:**
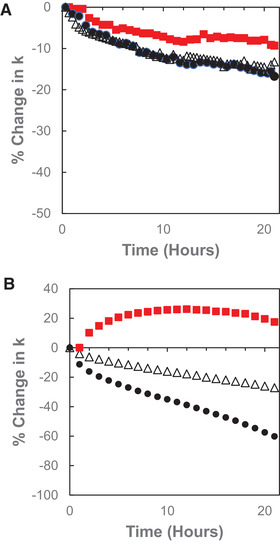
(A) Dependence of the percentage change in the retention factors of methyl paraben and thymidine 5′‐monophosphate (TMP) on the time exposed to 0.5% TFA at 60°C for BEH C_18_ AX, 1.7 μm (black circles – methyl paraben, red squares – TMP) and HSS T3, 1.8 μm (open triangles – methyl paraben) columns. (B) Dependence of the percentage change in the retention factors of propyl paraben and TMP on the time exposed to 0.02 M NaOH at 50°C for BEH C_18_ AX, 1.7 μm (black circles – propyl paraben, red squares – TMP) and CSH C_18_, 1.7 μm (open triangles – propyl paraben) columns

The stability of BEH C_18_ AX columns to acidic and basic mobile phases was assessed using accelerated tests employing harsh conditions chosen to show significant changes on a time scale of 1–2 days [15, 20]. For the acid stability test, the mobile phase contained 0.5% TFA (pH 1.3), and the test was further accelerated by using a temperature of 60°C. The retention factors of a neutral analyte (methyl paraben) and an acidic analyte (TMP) were monitored at regular intervals. A decrease in the retention factor is caused by hydrolysis of the bonded groups, with the hydrolysis products being removed by the mobile phase during the experiment [15]. Representative results for a BEH C_18_ AX column are compared to those for an HSS T3 column in Figure 5A. The rate of retention decrease for methyl paraben is very similar for the BEH C_18_ AX and HSS T3 columns, with a ca. 15% decrease observed after 22 h of exposure to the 0.5% TFA mobile phase. The rate of retention decrease for TMP on the BEH C_18_ AX column was found to be slightly slower, with a ca. 10% decrease after 22 h. This demonstrates the stability of the anion‐exchange groups. Based on these results, the suggested low pH limit for BEH C_18_ AX columns is 2, which is the same as the recommended low pH limit for HSS T3 columns [21].

Accelerated base stability tests were carried out using a mobile phase containing aqueous 0.02 M NaOH (pH 12.3) and a temperature of 50°C. The retention factor and efficiency of propyl paraben and TMP were monitored as a function of time. The rates of retention and efficiency loss for propyl paraben were similar. The retention results for a BEH C_18_ AX column are compared to those for a CSH C_18_ column in Figure 5B. The rate of retention decrease for propyl paraben is greater for the BEH C_18_ AX column than for the CSH C_18_ column, with a 50% change observed after 17 h, compared to 37 h. This difference is believed to be due to the higher particle surface area and the lower C_18_ surface concentration for BEH C_18_ AX relative to CSH C_18_ [16]. In contrast, the retention of the ionized acid TMP was found to increase over the first 12 h before slowly beginning to decrease. We speculate that the increase in retention of TMP results from greater accessibility of the anion exchange functionalities due to the loss of some of the C_18_ and endcap groups. This result also indicates the remarkable hydrolytic stability of the anion exchange groups in BEH C_18_ AX. Based on these results, the suggested upper pH limit for BEH C_18_ AX columns is 10.

To verify the upper pH limit, we carried out a stability study using a pH 10 mobile phase and a temperature of 30°C. A three‐component sample mixture was injected 340 consecutive times, with a run time of 1 h per injection. Two columns were tested. After 2 weeks of exposure to the pH 10 mobile phase, the averages and SD for the relative changes in retention time for resorcinol, guanosine‐5′‐monophosphate and nicotinamide were found to be −12.1% (SD 1.6%), −15.8% (SD 1.8%), and −7.2% (SD 0.8%) respectively. Relatively small decreases in efficiency (ca. 10%) were observed during this study. These results confirm that pH 10 is a reasonable upper limit for BEH C_18_ AX columns, when using a temperature of 30°C or lower. This is considerably higher than the upper pH limits of silica‐based RP/AX materials, which are typically 7.5 or lower (https://www.thermofisher.com/order/catalog/product/064984#/064984, https://www.sielc.com/primesep‐b.html). The extended upper pH limit of BEH C_18_ AX allows it to be used with a wider range of mobile phase pH values. For samples containing ionizable analytes, mobile phase pH has been demonstrated to be a key variable to use in optimizing RP separations [34, 35, 36, 37, 38].

### 3.7 Dependence of retention on mobile phase pH

**FIGURE 6:**
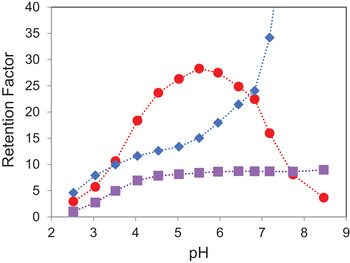
Dependence of retention factor on mobile phase pH for three ionizable compounds on a BEH C_18_ AX 1.7 μm 2.1 × 50 mm column: nicotinamide (purple squares), AMP (red circles), and procainamide (blue diamonds)

We investigated the dependence of retention on pH for a BEH C_18_ AX column by separating the mixture of Figure 2 using a series of mobile phases varying in pH, with a constant 20 mM concentration of ammonium formate. The retention factors of three of the analytes (AMP, nicotinamide, and procainamide) have a pronounced dependence on pH, as shown in Figure 6. As expected, the retention factors of the neutral analytes did not vary significantly with pH. For the two basic analytes, nicotinamide and procainamide, the retention factors were lower at pH values below their p*K*_a_, where they are protonated, then increase when the pH is above their p*K*_a_, where they are unprotonated. This is typical for basic compounds in RP chromatography [34, 36–38]. Very different behavior was observed for AMP, which has three ionizable groups: the two acidic P−OH groups and the basic adenine moiety. Based on the p*K*_a_ values in Figure 2, the ionic charge of AMP varies from 0 at approximately pH 2.5 to −2 at pH 7.2 and above. The increasing retention factor of AMP between pH 2.5 and 5.5 is attributed to its increasing negative charge. The decrease in its retention factor above pH 5.5 is due to the decreasing positive charge of the stationary phase [26].

## 4 CONCLUDING REMARKS

The Atlantis BEH C_18_ AX stationary phase exhibits mixed‐mode RP/weak anion‐exchange behavior, giving greater retention for anions (e.g., ionized acids) at pH values <9 compared to conventional RP materials and to charged surface hybrid stationary phases. Columns packed with this material exhibit high efficiencies, comparable to those of BEH C_18_ columns. We have found that this stationary phase is compatible with 100% aqueous mobile phases, reproducible from batch‐to‐batch and usable from pH 2 to 10. These characteristics are important for developing robust methods that may be used over long periods of time, such as those employed for quality control testing of pharmaceuticals. The hydrolytic stability of the BEH C_18_ AX stationary phase makes it well suited for LC/MS methods, as it causes minimal interference due to bonded phase hydrolysis products. One recent LC/MS application of BEH C_18_ AX columns involves the quantification of organic acids in foods [39].

The extended upper pH limit of the BEH C_18_ AX stationary phase relative to silica‐based mixed‐mode materials allows it to be used with a greater range of mobile phase pH values. This is a powerful tool for adjusting selectivity, since the ionization state of both the stationary phase and many analytes vary with pH. With an upper pH limit of 10, the BEH C_18_ AX stationary phase may be used at pH values above 8, where the positive surface charge is relatively low. This allows the use of pH to attenuate the retention of analytes that strongly interact by ion‐exchange, such as those containing multiple phosphate groups. This may be useful, for example, in methods employing a pH gradient, as recently demonstrated for LC/MS/MS analysis of nucleotides [40].

## CONFLICT OF INTEREST

The authors are employed by Waters Corporation, the manufacturer of the columns that were evaluated.
